# Enrichment of halotolerant hydrogen-oxidizing bacteria and production of high-value-added chemical hydroxyectoine using a hybrid biological–inorganic system

**DOI:** 10.3389/fmicb.2023.1254451

**Published:** 2023-08-29

**Authors:** Xiang Feng, Daichi Kazama, Sijia He, Hideki Nakayama, Takeshi Hayashi, Tomochika Tokunaga, Kozo Sato, Hajime Kobayashi

**Affiliations:** ^1^Department of Systems Innovation, Graduate School of Engineering, The University of Tokyo, Tokyo, Japan; ^2^Department of Environmental Science, Graduate School of Fisheries and Environmental Sciences, Nagasaki University, Nagasaki, Japan; ^3^Department of Regional Studies and Humanities, Faculty of Education and Human Studies, Akita University, Akita, Japan; ^4^Department of Environment Systems, Graduate School of Frontier Sciences, The University of Tokyo, Chiba, Japan; ^5^Frontier Research Center for Energy and Resource, Graduate School of Engineering, The University of Tokyo, Tokyo, Japan

**Keywords:** CO_2_ fixation, circular economy, enrichment, high salinity, hydroxyectoine, hydrogen-oxidizing bacteria, valuable products

## Abstract

Hybrid biological–inorganic (HBI) systems show great promise as CO_2_ conversion platforms combining CO_2_ fixation by hydrogen-oxidizing bacteria (HOB) with water splitting. Herein, halotolerant HOB were enriched using an HBI system with a high-ionic-strength medium containing 180 mM phosphate buffer to identify new biocatalysts. The reactors were inoculated with samples from saline environments and applied with a voltage of 2.0 V. Once an increase in biomass was observed with CO_2_ consumption, an aliquot of the medium was transferred to a new reactor. After two successive subcultures, *Achromobacter xylosoxidans* strain H1_3_1 and *Mycolicibacterium mageritense* strain H4_3_1 were isolated from the reactor media. Genome sequencing indicated the presence of genes for aerobic hydrogen-oxidizing chemolithoautotrophy and synthesis of the compatible solute hydroxyectoine in both strains. Furthermore, both strains produced hydroxyectoine in the reactors under the high-ionic-strength condition, suggesting the potential for new HBI systems using halotolerant HOB to produce high-value-added chemicals.

## 1. Introduction

The hybrid biological–inorganic (HBI) system represents a new variant of bioelectrochemical systems that uses hydrogen-oxidizing bacteria (HOB) and inorganic electrocatalysts to convert CO_2_ into value-added products. Typically, an HBI reactor comprises a single-chamber cultivation vessel containing a liquid medium and two electrodes (anode and cathode). Inorganic catalysts (usually metals) deposited on the electrode surfaces (or directly used as the electrode material) catalyze water electrolysis in the medium (electrolyte). HOB residing in the medium utilize the produced H_2_ and O_2_ as electron donors and acceptors, thereby reducing CO_2_ into cellular materials (i.e., products) via the Calvin–Benson–Bassham cycle. Such *in situ* water electrolysis mitigates the mass transfer limitation of hydrogen gas to the medium (Nangle et al., 2017). Utilizing genetically engineerable HOB (e.g., *Cupriavidus necator* H16 and *Rhodococcus opacus* DSM 43205) offers significant flexibility to the system and expands the range of possible products (Liu et al., 2016; Nyyssölä et al., 2021; Salusjärvi et al., 2022). To date, HBI systems have successfully produced chemicals including biofuels, fatty acids, biopolymers (and their precursors), biofertilizers, and dietary compounds (Torella et al., 2015; Liu et al., 2016; Brigham, 2019; Nyyssölä et al., 2021; Salusjärvi et al., 2022; Wu et al., 2022). However, for this technology to be a viable option for CO_2_ utilization, it is necessary to improve its energy and economic efficiencies.

The HBI system differs from conventional water electrolysis, which uses alkaline or acidic electrolytes, in that it must be operated at neutral pH to optimize bacterial growth. However, this condition leads to the oxygen evolution reaction being kinetically sluggish, resulting in a high activation overpotential and significant reactive oxygen species (ROS) generation as a side reaction (Prévoteau et al., 2020; Alqahtani et al., 2021). These issues can be largely overcome by the employment of a biocompatible catalyst system consisting of a self-healing cobalt phosphate (CoPi) anode and a cobalt–phosphorus (Co-P) alloy cathode, which can effectively catalyze water electrolysis at neutral pH and minimize the ROS generation, enabling high CO_2_ reduction energy efficiencies that exceed those of natural photosynthetic systems (Liu et al., 2016). However, the low concentration of ions at neutral pH reduces the conductivity of the electrolyte, resulting in a relatively high internal resistance in the reactor. Increasing the ionic strength of the medium can increase the conductivity, thereby facilitating electrochemical reactions (Bian et al., 2020; Jourdin and Burdyny, 2021; Liu et al., 2023). Water splitting with Co-P|CoPi catalysts is promoted by increasing the concentration of the phosphate buffer from 36 to 108 mM (Liu et al., 2016); however, previous studies on HBI systems have mainly used media with low-ionic-strength (i.e., phosphate buffer concentration of ≤36 mM) owing to the salt sensitivity of the biocatalysts used (e.g., *C. necator* and *Xanthobacter autotrophicus*) (Torella et al., 2015; Liu et al., 2016). Thus, using halotolerant HOB as the biocatalyst could potentially address this limitation (Barbosa et al., 2021b).

In other variants of bioelectrochemical systems, such as microbial fuel cells and microbial electrosynthesis systems, it has been suggested that the use of halotolerant bacteria allows the system to operate under higher ionic conditions, thereby reducing internal resistance and improving performance. Halophilic CO_2_-fixing microbial communities have been used as biocatalysts in microbial electrosynthesis to produce acetate (Alqahtani et al., 2019, 2021; Kiran et al., 2023; Zhang et al., 2023), exhibiting an improvement in efficiency with high-ionic-strength electrolytes. Furthermore, halotolerant bacteria are known to accumulate compatible solutes, such as ectoine, hydroxyectoine, glycine betaine, and proline betaine, to counteract osmotic stresses. These compounds are commercially relevant because they have potential uses as moisturizers and protein stabilizers in the cosmetics and food industries (Liu et al., 2019; Sekar and Kim, 2020; Cantera et al., 2022).

This study aimed to isolate and characterize halotolerant HOB as new biocatalysts for the HBI system. HBI reactors can be used to enrich HOB suitable for conditions specific to the system, such as variable H_2_ and O_2_ concentrations, the presence of inorganic catalysts, ROS, and specific medium compositions with limited nutrient availability and ionic strengths. In our previous study, environmental samples were inoculated into HBI system reactors using Co-P|CoPi catalysts and a medium with moderate ionic strength (108 mM phosphate buffer) to enrich HOB. After enrichment via serial fed-batch operations, members of *Acidovorax*, *Hydrogenophaga*, *Mycolicibacterium*, and *Xanthobacter* were enriched and readily isolated (Feng et al., 2023). In the present research, HOB with high salt tolerance were enriched from saline environments by using an HBI system with a high-ionic-strength medium containing 180 mM phosphate buffer, the salinity of which is equivalent to seawater. The resulting isolates were shown to synthesize hydroxyectoine in the reactor, presenting a proof of concept for a new HBI system to produce high-value-added extremolytes from CO_2_ under high-ionic-strength conditions.

## 2. Results

### 2.1. Enrichment of halotolerant HOB using the HBI system under a high-ionic-strength condition

To confirm the reactor preparations, the Faradaic efficiency of the H_2_ evolution reaction (HER) was measured in several representative reactors without any microbial inoculation. The obtained Faradaic efficiencies of HER were approximately 40%–60%, which were lower than those reported previously (96%: Liu et al., 2016). It has to be noted that this difference in Faradic efficiencies could be due to the distinct experimental setup used in those studies. The Faradic efficiency was measured in a two-chamber electrochemical cell using 100 mM KPi buffer as the electrolyte in the prior study (Liu et al., 2016), while this study used the single-chamber reactor with the high-ionic-strength medium as the electrolyte (the same setting as the following enrichment experiments). Thus, the observed lower Faradaic efficiencies could likely be attributed to hydrogen recycling, where H_2_ produced at the cathode was abiotically oxidized at the anode. Additionally, the presence of possible side reactions, such as interactions between metal ions in the medium and the electrodes, may have contributed to the reduced efficiencies. Yet, the results confirmed that the reactor performance was sufficient for the enrichment purpose.

**Figure 1:**
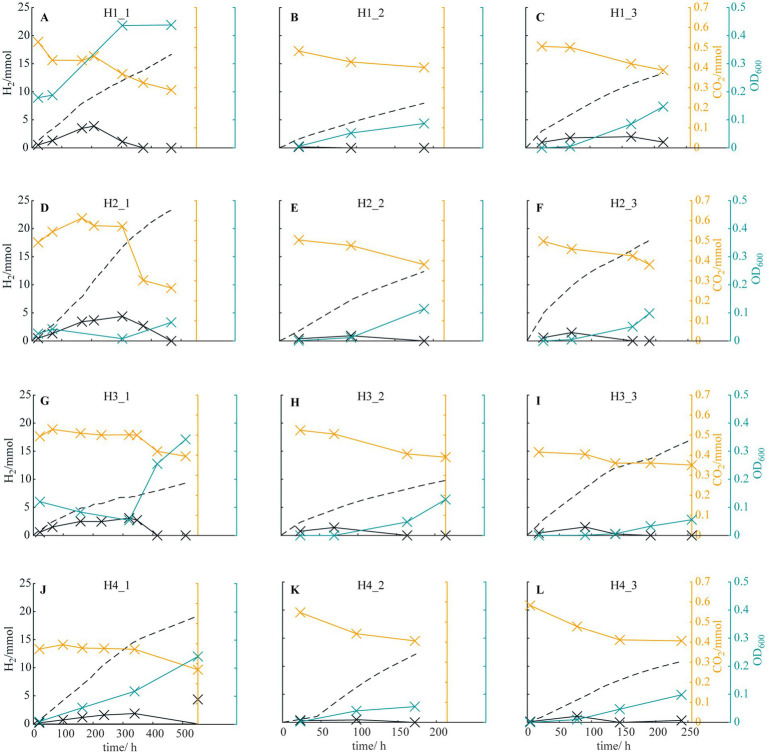
Theoretical H_2_ production deriving from the current (dashed black line), H_2_ (solid black line) and CO_2_ measured (orange line) in the headspace and the optical density OD_600_^1cm^ of the medium (blue line) for reactors **(A)** H1_1, **(B)** H1_2, **(C)** H1_3, **(D)** H2_1, **(E)** H2_2, **(F)** H2_3, **(G)** H3_1, **(H)** H3_2, **(I)** H3_3, **(J)** H4_1, **(K)** H4_2, and **(L)** H4_3.

To enrich halotolerant HOB, environmental samples from brackish/marine environments were inoculated into HBI reactors using a high-ionic-strength medium containing 180 mM phosphate buffer as the culture medium/electrolyte (Table 1). Then, the reactors were operated under the same condition (except the initial inocula). The pH of the medium was around 7.1 at the beginning of operations and no fluctuation was observed. To estimate HOB growth in the reactors, H_2_ and CO_2_ concentrations in the reactor headspace, the medium turbidity, and theoretical H_2_ production (estimated from the current flowing through the reactor circuit) were monitored (Figure 1). When H_2_ and CO_2_ consumption were observed markedly, a few microliters of reactor medium were inoculated into a new reactor to start the next enrichment cycle. For each inoculum source, three enrichment cycles were performed. In general, initial lag periods with various durations were observed in the reactors, particularly in reactors H2_1 and H3_1. After lag periods, CO_2_ and H_2_ consumption were initiated simultaneously, and an increase in medium turbidity was observed in all reactors. Furthermore, the durations until the H_2_ concentration in the headspace became undetectable were shortened in later enrichment cycles (approximately 400 and 200 h for the first and third cycles, respectively), indicating successful enrichment of halotolerant HOB in the reactors under a high-ionic-strength condition.

**Table 1:** Settings for the enrichments.

Reactor	Inoculum source	Enrichment cycle	Enrichment period (*h*)
H1_1	Brackish water sampled from a brackish river, Shiokawa (26.65583N, 127.98804E) in Nago, Okinawa, Japan on June 2022	1st	467
H1_2	Liquid medium from reactor H1_1	2nd	188
H1_3	Liquid medium from reactor H1_2	3rd	214
H2_1	Beach sediment sampled from a coral beach (26.60218N, 127.94125E) in Nago, Okinawa, Japan on June 2022	1st	467
H2_2	Liquid medium from reactor H2_1	2nd	188
H2_3	Liquid medium from reactor H2_2	3rd	190
H3_1	Bay sediment sampled from 15 m below sea level (35.157769N, 139.609800E) at Aburatsubo Bay in Miura, Kanagawa, Japan on June 2022	1st	510
H3_2	Liquid medium from reactor H3_1	2nd	214
H3_3	Liquid medium from reactor H3_2	3rd	256
H4_1	Estuarine sediment collected from a coastal river (34.056594N, 132.872074E) in Imabari, Ehime, Japan on November 2021	1st	551
H4_2	Liquid medium from reactor H4_1	2nd	172
H4_3	Liquid medium from reactor H4_2	3rd	240

### 2.2. Composition of the enriched communities in the HBI system

**Figure 2:**
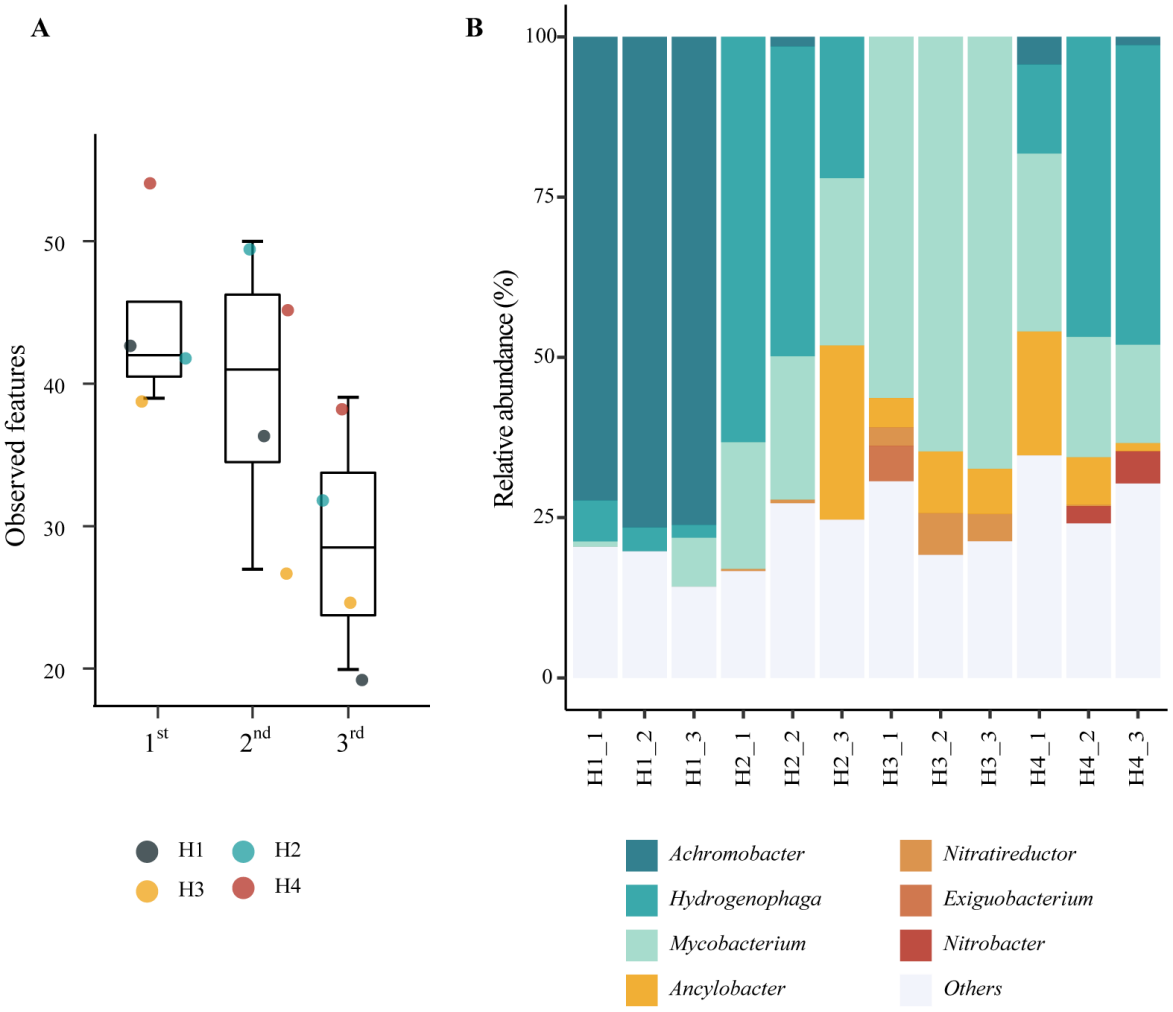
Phylogenetic analyses of microbial communities enriched in the HBI system. **(A)** Alpha-diversity indices estimating ASV richness according to enrichment cycles (*p* = 0.08, Kruskal-Wallis test). The tops and bottoms of boxes represent the 75th and 25th percentiles, respectively. The upper and lower whiskers extend to data points no more than 1.5 times the interquartile range from the upper and lower edges of the box, respectively. **(B)** Relative abundance at the genus level according to the inoculum sources. Taxa representing <5% of sequences in all samples are grouped in the “Others” category.

The phylogenetic diversity of bacterial communities enriched in the reactors was assessed using 16S rRNA gene amplicon sequencing. Alpha diversity was calculated using the amplicon sequence variant (ASV) data (Figure 2A). The number of ASVs observed in the third cycle was markedly lower than that observed in the first cycle, indicating that specific bacteria were selected through the enrichment cycles. Principal coordinate analysis based on Bary–Curtis distances (Supplementary Figure S1) showed a clear distinction between reactors H1, H3, and reactors H2 with H4, indicating that the origin of the initial inoculum significantly affects the resulting microbial enrichments.

Taxonomic annotation of the amplicon sequences revealed high relative abundances of seven genera in the reactors (Figure 2B). The genus *Achromobacter* was the dominant genus in the reactors of series H1, accounting for 72.32%, 76.54%, and 76.12% of the sequences from reactors H1_1, H1_2, and H1_3, respectively. In the reactors of the H2, H3, and H4 series, the genus *Mycolicibacterium* was highly abundant. In particular, the relative abundance of *Mycolicibacterium* increased after the enrichment cycles in series H2 (from 19.78% in H2_1 to 26.09% in H2_3) and H3 (from 56.35% in H3_1 to 67.38% in H3_3). *Mycolicibacterium* also showed the second highest abundance in reactor H4_3 (15.37%) behind *Hydrogenophaga*, which itself showed an increase in abundance in series H4 (from 13.89% in H4_1 to 46.72% in H4_3) and a high abundance in series H2 (63.25% in H2_1). In our previous study, HOB species of the genera *Mycolicibacterium* and *Hydrogenophaga* were enriched in HBI systems using a medium with 108 mM phosphate buffer (Feng et al., 2023). Additionally, three putative nitrate-reducing bacteria, including *Ancylobacter* (increased to 27.15% in H2_3), *Nitratireductor* (increased to 4.23% in H3_3), and *Nitrobacter* (increased to 5.02% in H4_3), showed an increase in abundance with increasing enrichment cycles.

### 2.3. Isolation of halotolerant HOB

After the third enrichment cycle, HOB were isolated from the culture media through single colony isolation, resulting in the successful isolation of 13 strains as follows: strains H1_3_1 and H1_3_2 from reactor H1_3; strains H2_3_1 and H2_3_2 from reactor H2_3; strains H3_3_1, H3_3_2, H3_3_3, and H3_3_4 from reactor H3_3; and strains H4_3_1, H4_3_2, H4_3_3, H4_3_4, and H4_3_5 from reactor H4_3 (Supplementary Figure S2). All 13 isolates were capable of chemolithoautotrophic growth in the high-ionic-strength liquid medium with an H_2_/CO_2_/O_2_ (80:10:10) gas mixture. Additionally, chemolithoautotrophic growth in the low-ionic-strength medium (containing 36 mM phosphate buffer) and aerobic organotrophic growth (in Luria–Bertani medium) were also observed (data not shown). Therefore, all isolates were identified as facultative aerobic HOB that were capable of thriving under a high-ionic-strength condition.

The 16S rRNA sequences of the two isolates from reactor H1_3 were nearly identical to each other and had high similarity with species of genus *Achromobacter*. The other 11 isolates from reactors H2_3, H3_3, and H4_3 were also highly similar to each other and closely related to genus *Mycolicibacterium*. This result is consistent with the phylogenetic compositions of microbial communities enriched in the reactors, wherein *Achromobacter* and *Mycolicibacterium* showed high relative abundance. Two representative isolates, strains H1_3_1 and H4_3_1, were selected for further analyses.

### 2.4. Analysis of whole genome sequences of the selected isolates

To better understand the mechanisms underlying the chemolithoautotrophy, hydrogen oxidization, and halotolerance of the isolates, the whole genome sequences of representative isolates were determined. The strains H1_3_1 and H4_3_1 each contain a single circular chromosome of approximately 7.1 and 7.6 Mbp, which showed overall similarities of 70.7% and 92.4% (in the DNA–DNA hybridization values) with the type strains of *A. xylosoxidans* LMG 1863 (GCF_000508285.1) and *M. mageritense* JCM 12375 (GCF_010727475.1), respectively, indicating that the isolates belong to these species.

Functional annotation of the genomes revealed that both strains encode genes for a complete Calvin–Benson–Bassham cycle as well as genes for a complete tricarboxylic acid cycle and oxidative phosphorylation, indicating their capability of carbon fixation and aerobic respiration. The genome of *A. xylosoxidans* strain H1_3_1 encodes two O_2_-tolerant hydrogenases, a Group 1d [NiFe]-hydrogenase and a Group 2b [NiFe]-hydrogenase, which are a membrane-bound respiratory H_2_-uptake hydrogenase and H_2_-sensing regulatory hydrogenase, respectively. The genome of *M. mageritense* strain H4_3_1 encodes one O_2_-tolerant hydrogenase, a Group 2a [NiFe]-hydrogenase, which can use O_2_ as the terminal electron acceptor. Both genomes also contain homologs of *hyp* genes, encoding proteins for the maturation of [NiFe]-hydrogenases (Greening et al., 2016; Fan et al., 2022).

Notably, a complete set of genes for ectoine/hydroxyectoine biosynthesis (*ectA*, *ectB*, *ectC*, and *ectD*) are found in both genomes, suggesting that these HOB strains cope with the high-ionic-strength environment in the reactor by producing the compatible solutes.

### 2.5. Physiological and chemotaxonomical characteristics of *Achromobacter xylosoxidans* strain H1_3_1

The chemolithoautotrophic growth of *M. mageritense* had been demonstrated in our previous study. On the other hand, so far chemolithoautotrophic metabolism has never been reported in *A. xylosoxidans*, while two members of genus *Achromobacter*, *A. ruhlandii* LMG 1866 and *A. veterisilvae* LMG 30378, have been reported to be HOB (Packer and Vishniac, 1955; Dumolin et al., 2020). Thus, the strain H1_3_1 was characterized and compared with the type strain of *A. xylosoxidans*.

*A. xylosoxidans* strain H1_3_1 were motile and small bacilli with a length of 1.0–2.0 μm and a width of 0.5–0.6 μm. Colonies were convex, nontransparent, cream color, with smooth margins and a diameter under 1 mm. Cells were Gram negative and spore formation was not detected. Growth was observed at all temperatures tested (28°C, 37°C, and 45°C), while growth at 45°C was relatively weak. Catalase and oxidase activities were detected. No fermentation/oxidization of glucose was observed and no acid/gas was produced from glucose. Growth on the MacConkey media tested positive. Nitrate reduction activity was detected. Glucose, potassium gluconate, capric acid, adipic acid, malic acid, trisodium citrate, and phenylacetic acid are assimilated. No indole production from L-tryptophane, fermentation of glucose, arginine dehydrolase, urease or β-galactosidase activities, esculin or gelatin hydrolysis, or assimilation of arabinose, mannose, mannitol, N-acetyl-glucosamine, and maltose were observed. Activities of esterase, leucine arylamidase, and acid phosphatase were detected. Weak activities of alkaline phosphatase and Naphthol-AS-BI-phosphohydrolase were also detected. No activity of esterase lipase, lipase, valine arylamidase, cystine arylamidase, trypsin, α-chymotrypsin, α-galactosidase, β-galactosidase, β-glucuronidase, α-glucosidase, β-glucosidase, N-acetyl-β-glucosaminidase, α-mannosidase or α-fucosidase was detected. Overall, the physiological characteristics of the strain H1_3_1 were highly similar to those of *A. xylosoxidans* LMG 1863, the type strain which is not HOB (Supplementary Table S2). Moreover, the cellular fatty acids composition of stain H1_3_1 showed overall similarity with that of the type strain (Vandamme et al., 2013) (Supplementary Table S1). Furthermore, the respiratory quinone composition was 99% ubiquinone 8 (Q8), 0.5% Q7, and 0.5 Q9, which are also similar to the type strain (Yabuuchi et al., 1998).

Interestingly, in the genome of *A. xylosoxidans* strain H1_3_1, above mentioned genes for hydrogenases and their maturation proteins (*hox* and *hyp* genes) and enzymes of the Calvin–Benson–Bassham cycle (*cbb* genes) are located just proximal to each other (Supplementary Figure S3). Moreover, the *hox*-*hyp*-*cbb* cluster is flanked by two identical insertion sequences (IS*256*) with an ORF encoding putative integrase next to one of them, suggesting that the *hox*-*hyp*-*cbb* cluster is encoded on a transposable element. Thus, these results suggest that the strain H1_3_1 is a noble HOB strain of *A. xylosoxidans*, which likely acquired the *hox*-*hyp*-*cbb* cluster via lateral gene transfer.

### 2.6. Hydroxyectoine production of the isolated HOB in the HBI system

**Figure 3:**
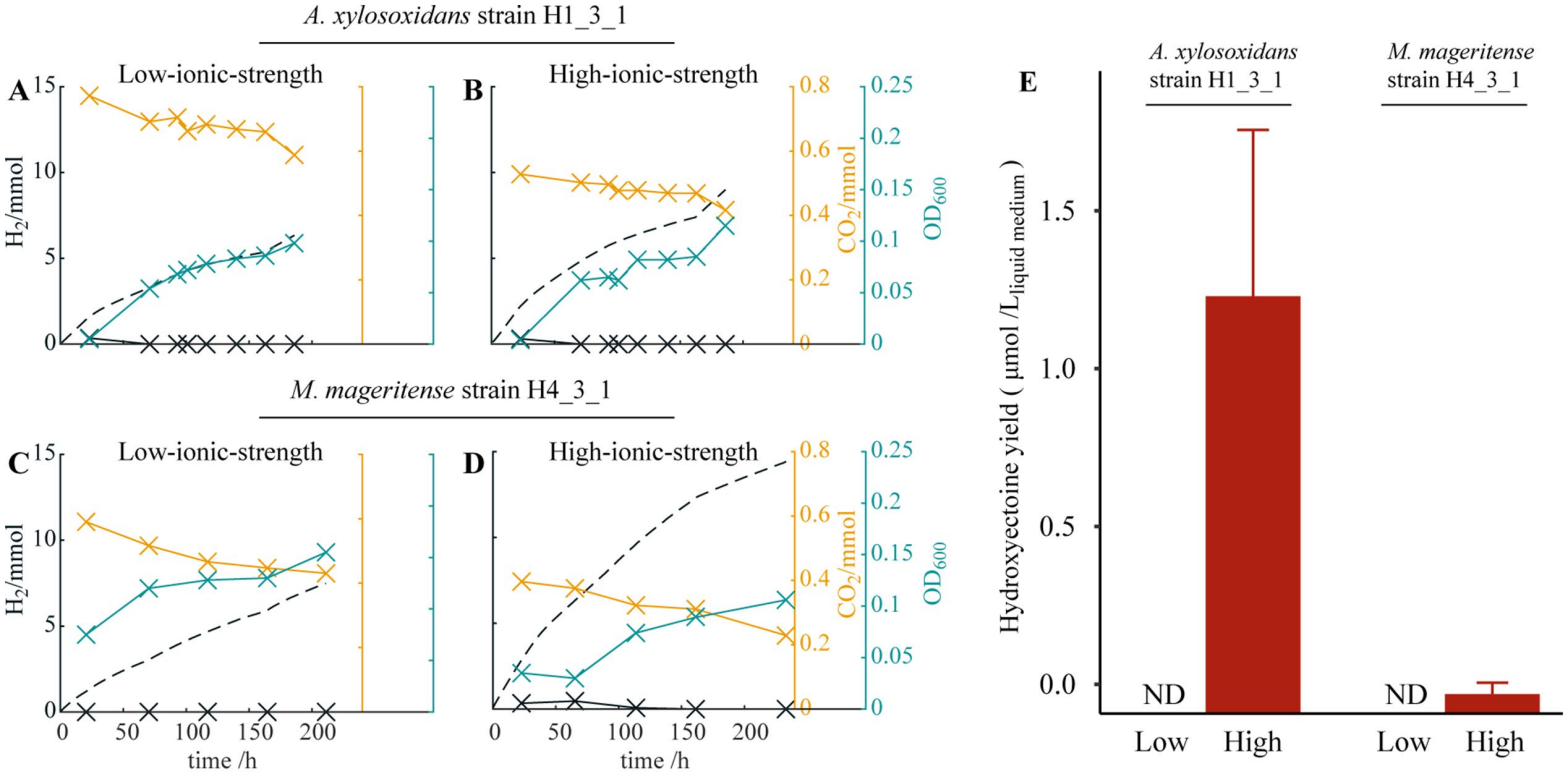
Theoretical H_2_ production deriving from the current (dashed black line), H_2_ (solid black line) and CO_2_ measured (orange line) in the headspace, and the optical density OD_600_^1cm^ of the medium (blue line) for **(A)** *A. xylosoxidans* strain H1_3_1 in low-ionic-strength medium, **(B)** *A. xylosoxidans* strain H1_3_1 in high-ionic-strength medium, **(C)** *M. mageritense* strain H4_3_1 in low-ionic-strength medium, and **(D)** *M. mageritense* strain H4_3_1 in high-ionic-strength medium; **(E)** Average intracellular hydroxyectoine yield at different salt concentrations. ND represents not detected.

To examine the production of the compatible solutes in the HBI system, *A. xylosoxidans* strain H1_3_1 and *M. mageritense* stain H4_3_1 were, respectively, cultivated in the reactors containing high-ionic-strength medium or low-ionic-strength medium for comparison. EIS confirmed that internal resistance of the reactor with high-ionic-strength medium was smaller than that of the reactor with low-ionic-strength medium (Supplementary Table S3). No bacterial growth was observed in abiotic controls or open-circuit biotic controls (data not shown). The two HOB strains were able to grow under both conditions, as shown in Figures 3A–D. Following cultivations in the reactors, the microbial cells were harvested, and ectoine/hydroxyectoine were extracted. Notably, accumulations of hydroxyectoine were detected in strains H1_3_1 and H4_3_1 cultivated in the reactor under the high-ionic-strength condition (Figure 3E), while accumulations of ectoine were below the detection limit (data not shown). The yields of hydroxyectoine were 1.32 μmol/L_liquid medium_ for *A. xylosoxidans* strain H1_3_1 and 0.06 μmol/L_liquid medium_ for *M. mageritense* strain H4_3_1. On the other hand, no ectoine/hydroxyectoine was detected in the cells cultivated in the reactors with the low-ionic-strength medium, suggesting that hydroxyectoine synthesis is induced under a high-ionic-strength condition and supports the notion that the compatible solute plays a crucial role in the halotolerance of HOB isolates.

## 3. Discussion

The HBI reactor with the high-ionic-strength media was used to screen HOB with halotolerant properties, resulting in the isolation of *A. xylosoxidans* strain H1_3_1 and *M. mageritense* strain H4_3_1. Both strains grew in the HBI reactor under a high-ionic-strength condition and produced the compatible solute hydroxyectoine. At the same time, in accordance with the smaller internal resistance (Supplementary Table S3), the reactor with the high-ionic-strength medium exhibited a larger current flow, indicating a stronger water electrolysis reaction. However, the growth of these HOB strains was not improved a lot relative to that under a low-ionic-strength condition. Therefore, the use of a high-ionic-strength medium and halotolerant HOB did not improve the CO_2_ reduction energy efficiency of the HBI system in terms of biomass accumulation. It is plausible to assume that this is at least partly due to the adaptation responses of the bacteria to osmotic stress. Our results show that the high-ionic-strength condition induces the production of hydroxyectoine, which is synthesized from aspartate and can limit the production of other cellular materials. However, the high-ionic-strength condition did not severely impair the growth of strains H1_3_1 and H4_3_1 in the HBI system. Our findings suggest the potential use of a “high-ionic-strength HBI system” using halotolerant biocatalysts that can convert CO_2_ into high-value-added chemicals such as hydroxyectoine/ectoine. Additionally, homologs of genes (*ostA* and *ostB*) involved in trehalose synthesis were identified in the genome of *M. mageritense* strain H4_3_1, suggesting its potential use in the synthesis of trehalose from CO_2_.

Thus, this study provides a “proof of concept” for the new system using halotolerant HOB. This system might be more expensive than the conventional HBI systems The high-ionic-strength media require more chemical components, increasing the cost. Materials with higher salt resistance will be required for the reactor and tubing. Additional facilities for treating high-ionic-strength wastewater should also be considered. Yet, the economic benefits derived from high-value products could potentially compensate for these additional costs. Moreover, other value-added products (such as vitamins, triacylglycerols, and protein) can be harvested as by-products of the extremolytes (Shteĭnberg and Datsiuk, 1985; Zhang et al., 2019; Barbosa et al., 2021b). Additionally, members of halotolerant HOB could serve as a means of removing pollutants, such as phosphate ions, from wastewater (Barbosa et al., 2021a). However, several issues must be addressed before its practical application. First, the biological safety of the biocatalysts should be confirmed before considering their large-scale applications. Some *A. xylosoxidans* and *M. mageritense* strains are known to be opportunistic pathogens (Busse and Auling, 2015; Magee and Ward, 2015), however, it is less likely that the isolates are pathogenic, as they originated from non-host environments and no obvious gene for pathogenesis (such as pathogenic islands) was found in their genomes. Yet, to be on the safe side, further screening of nonpathogenic halotolerant HOB might be helpful. However, HOB strains closely related to *M. mageritense* strain H4_3_1 were isolated from two other reactors (H2_3 and H3_3) and a beach sediment sample in our previous study (Feng et al., 2023), suggesting that mycolicibacterial HOB are widely distributed in saline environments and can be easily propagated in HBI systems. Thus, samples from a wider range of environments can be screened using HBI systems (Fu et al., 2015; Kobayashi et al., 2021). Alternatively, genes involved in halotolerance (e.g., *ectABCD*) could be introduced into a genetically modifiable HOB, such as *C. necator*.

Second, like other HBI systems, further improvements are required in system design, such as optimizing the operational conditions and reactor configurations (Li et al., 2017, 2019; Li J. et al., 2020). Specifically, the ion strength of the medium must be optimized to maximize the yield of hydroxyectoine. Very recently, it has been reported that five HOB species (*Hydrogenibacillus schlegelii*, *Hydrogenovibrio marinus*, *Pseudonocardia autotrophica*, *Pseudomonas dioxanivorans* and *Rhodococcus opacus*) can produce ectoine/hydroxyectoine from CO_2_ under H_2_/air/CO_2_ atmospheres, where gaseous H_2_, air, and CO_2_ were directly supplied to HOB (Cantera et al., 2023). In our study, the yields of hydroxyectoine in the HBI system were generally smaller than those reported in the above-mentioned research, suggesting that the supply of H_2_ and O_2_ is a limiting factor in the HBI system. It must be noted that the reactor system used here was designed for the enrichment of HOB from environmental samples and showed relatively low Faradaic efficiencies. Moreover, to prevent contamination, the reactors were autoclaved (which could potentially damage the deposited catalysts) and operated in batch mode (which is not optimal for bacteria growth). Therefore, there is potential for further improvement of reactor efficiency. Additionally, the current flow in the reactor inoculated with *A. xylosoxidans* strain H1_3_1 gradually attenuated after operations lasting more than 4 days. Microscopic observations showed that the bacteria were attached to the electrode surfaces, particularly the cathode surface, suggesting that the cells attached to the electrodes likely inhibited the activity and self-healing of the catalysts (Supplementary Figure S4). Consistent with this finding, the genome of *A. xylosoxidans* strain H1_3_1 encodes genes likely involved in cell adhesion and biofilm development (such as *pag* and *flg* gene clusters). To mitigate these issues, the reactor/electrode configuration could be optimized (e.g., by adding separators), and new electrode materials/catalysts could be employed (Li Q. et al., 2020). More generally, basic studies for understanding the physiological responses/adaptations of HOB to the reactor environments are still required for the successful scale-up of HBI systems.

## 4. Conclusion

In this study, an HBI system with a high-ionic-strength medium was used to screen HOB with halotolerant properties, resulting in the isolation of *A. xylosoxidans* strain H1_3_1 and *M. mageritense* strain H4_3_1. Both strains grew in the HBI reactor under a high-ionic-strength condition and produced the compatible solute hydroxyectoine, presenting a promising opportunity for future applications involving the conversion of CO_2_ into high-value products using halotolerant HOB. In our future study, the yield of hydroxyectoine will be maximized by optimizing the system design, including the medium compositions, such as the ion strength and pH, as well as exploring different operation modes, such as continuous, fed-batch, and induced production by salt addition. Furthermore, the electrode materials and methods for catalyst deposition will be investigated, and the reactor design/configuration/control will be examined to ensure the most favorable conditions for hydroxyectoine production.

## 5. Materials and methods

### 5.1. Inoculum sources

Four samples were collected from saline environments and used as the initial microbial sources (Table 1). The samples were stored in 50 mL sterile tubes at 4°C until inoculation.

### 5.2. Electrode configuration

Synthesis of CoPi anode and Co-P cathode was operated by electrochemically depositing the catalysts on a 2 × 4 cm SUH316 stainless-steel mesh using previously described methods (Paseka and Velicka, 1997; Kanan and Nocera, 2008; Liu et al., 2016; Feng et al., 2023). The deposition process was performed using an electrochemical measurement system (HZ-7000, Hokuto Denko, Japan) referred to Ag/AgCl electrode. Following deposition, the electrodes were rinsed with deionized water.

### 5.3. HBI system reactors construction and operation

Borosilicate glass cells (220 mL) equipped with the CoPi anode and Co-P cathode fabricated in Section 5.2 was used as the HBI system reactors. The high-ionic-strength medium, a modification of the minimal medium (Feng et al., 2023) that contains 180 mM phosphate buffer (33.7 g/L Na_2_HPO_4_·7H_2_O and 7.5 g/L KH_2_PO_4_), was used as the culture medium. For experiments with a low-ionic-strength medium, the buffer strength of phosphate was decreased to 36 mM with 6.74 g/L Na_2_HPO_4_·7H_2_O and 1.5 g/L KH_2_PO_4_. The reactors and medium were sterilized separately by autoclaving. Before inoculation, 100 mL of the sterilized medium was added to the reactor in an aseptic manner.

For the initial enrichment cycle, approximately 10 mL of an environmental sample was filtered through a 0.45 μm nitrocellulose filter (Nalgene Nunc International, United States) in an analytical filter unit under vacuum conditions. The residue trapped on the filter was rinsed three times with a sterilized medium and inoculated into the reactor. After sealing the reactor with a butyl rubber plug and an aluminum closure, the ambient gas in the headspace was replaced with an 80:20 mixture of N_2_ and CO_2_. The reactor was kept at 30°C, continuously agitated with a magnetic stirrer, and subjected to a constant 2.0 V voltage via a power supply (3645A, Array Electronics, China) during the incubation. For the second and third enrichment cycles, a few microliters of the liquid medium from the previous enrichment cycle were collected using a sterilized inoculating loop and inoculated into a freshly prepared reactor. Thus, the experimental conditions of reactor series H1, H2, H3, and H4 were identical except the initial inocula (i.e., four different environmental samples: Table 1).

### 5.4. Analytical measurements and calculations

The voltage of a 1.0 Ω resistance across the electrodes was monitored with a multimeter (34970A, Agilent Technologies, United States). The electrical current flowing through the circuit was calculated based on Ohm’s law. The headspace pressure was monitored using a pressure sensor (APC40, Keyence, Japan). Gas chromatography with a thermal conductivity detector (GC-2014, Shimadzu, Japan) and argon as the carrier gas was used to analyze the gas composition. To monitor microbial growth, the optical density (OD^600^_1cm_) of the culture medium was measured using a spectrophotometer (Ultrospec 6,300 pro, GE Healthcare, United States). Electrochemical impedance spectroscopy (EIS) was employed to determine the internal resistance of the HBI reactor as described in Kim et al. (2021). The measurement was performed using an electrochemical measurement system (HZ-7000). The solution resistance (*R*_ohm_) was determined using a two-electrodes setup, where Co-P alloy served as the working electrode and CoPi as the counter electrode. To measure the charge transfer resistance of each electrode (*R*_act_anode_ and *R*_act_cathode_), a three-electrode setting was used with Ag/AgCl as a reference, then the values were determined from the semi-circular from the Nyquist plot. The total internal resistance *R*_int_ was then calculated as the summation of the charge transfer resistances and the solution resistance.

The theoretically produced hydrogen *n*_H2___theoretical_ (mol) was determined according to Equation 1:


(1) $$n_{{}^{H_{2}\_\mathrm{theoretical}}}=\overset{T}{\underset{t}{\sum }}\left(I\left(t\right)/\left(2*F\right)\right)$$

*I*(*t*) is the current (A) at sampling time interval *t* (sec), *T* is the experiment duration (sec), and *F* is Faraday’s constant (96,485 C/mol).

### 5.5. Phylogenetic characterization of microbial communities in reactors

Microbial cells were extracted from approximately 50 mL of the reactor medium using vacuum filtration through a 0.45 μm nitrocellulose filter in an analytical filter unit (Nalgene Nunc International). DNA extraction was operated following the protocol described in Feng et al. (2023). 16S rRNA genes were amplified by PCR using the extracted DNA as the template and primers U789F (5′-TAGATACCCBGGTAGTCC-3′) and U1068R (5′-CTGACGRCRRCCATGC-3′) (Baker et al., 2003) as described previously (Feng et al., 2023). The resulting PCR products were subjected to library preparation using Nextera XT DNA Library Prep Kits (Illumina, United States) according to the manufacturer’s instructions, and the resulting amplicons were sequenced on a MiSeq system (Illumina) at Biken Biomics (Japan). The resulting raw reads were processed on the QIIME2 platform (version 2021.4.0) using DADA2 to cluster them into amplicon sequence variants (ASVs) (Callahan et al., 2016; Bolyen et al., 2019). The alpha diversity (observed features) was calculated, and the taxonomy was assigned using the Blast+ algorithm against the Sliva (138 SSURef NR99) 16S rRNA database (Camacho et al., 2009; Robeson et al., 2021).

### 5.6. Isolation of HOB from the reactor medium

The medium from the reactors at the end of the third enrichment cycle was collected and serially diluted to 1:100,000 with the sterilized medium. Subsequently, 100 μL of each diluted sample was spread onto plates containing the high-ionic-strength medium solidified with 1% (w/v) gellan gum. The plates were cultured under an 80:10:10 H_2_/CO_2_/O_2_ atmosphere at 30°C. After around one week of incubation, colonies were isolated by picking, spreading, and cultivating them on new plates. This procedure was repeated thrice to guarantee the purity of the isolates.

The nearly full-length 16S rRNA gene sequences were obtained following the procedures described in Feng et al. (2023), using the primer pairs 8F (5′-AGAGTTTGATYMTGGCTCAG-3′) and 1492R (5′-CGGYTACCTTGTTACGACTT-3′) (Grabowski et al., 2005). The amplified fragment sequences were determined using standard Sanger sequencing at Macrogen Japan (Japan) and compared against the EzBioCloud database (Yoon et al., 2017).

### 5.7. Genome sequencing, genome assembly, and gene annotation

The HOB isolate was propagated in 20 mL of the high-ionic-strength medium under an H_2_/CO_2_/O_2_ atmosphere (80:10:10) at 30°C for two days and gathered via centrifugation. Genomic DNA was extracted from the cell pellet using Genomic-tip 20/G (Qiagen) with the Genomic DNA Buffer Set (Qiagen) for *Achromobacter* species or the MasterPure Gram Positive DNA Purification Kit (Epicentre, USA) for *Mycolicibacterium* species. The DNA template library was constructed using the SMRTbell gDNA Sample Amplification Kit (PacBio, USA) and SMRTbell Express Template Prep Kit ver.2.0 (PacBio) and bound to Sequel II polymerase 2.2 (PacBio) using the Sequel II Binding Kit 2.2 (PacBio). Sequencing was performed using the Sequel IIe platform (PacBio) at Bioengineering Lab (Japan). After trim of the overhand adapter and removal of low-quality and short reads, the resulting high-quality reads were assembled using Flye (ver.2.9.1-b1780) (Kolmogorov et al., 2019). Annotation was performed using Prokka (ver. 1.14.5) to identify relevant features and generate protein sequences (Seemann, 2014), which were further analyzed using eggNOG-mapper (ver. 2.1.9) against the Kyoto Encyclopedia of Genes and Genomes (KEGG) database (Huerta-Cepas et al., 2019; Cantalapiedra et al., 2021). Digital DNA–DNA hybridization values were estimated using the Genome-to-Genome Distance Calculator (ver. 3.0) (Meier-Kolthoff et al., 2022). Hydrogenase genes were classified using HydDB (Greening et al., 2016; Sondergaard et al., 2016).

### 5.8. Physiological and chemotaxonomical characterization of *Achromobacter xylosoxidans* strain H1_3_1

*Achromobacter xylosoxidans* strain H1_3_1 was grown on nutrient agar (Oxoid) at 30°C under aerobic conditions for 2 days and observed using a light microscope (Olympus BX50F4). Conventional phenotypic tests were performed as described in Barrow and Feltham (1993). The activity of constitutive enzymes and other physiological properties were determined using the API 20NE and API ZYM microtest systems (bioMérieux) according to the manufacturer’s instructions. For cell fatty acid analysis, strain H1_3_1 was cultivated on TSBA (Becton Dickinson) at 30°C under aerobic conditions for 24 h. The cells were freeze-dried, and fatty acid methyl esters were extracted and analyzed using the Sherlock Microbial Identification System (ver.6.0) according to the manufacturer’s procedures. Analysis of respiratory quinones was carried out by Deutsche Sammlung von Mikroorganismen und Zellkulturen GmbH (DSMZ, Germany). Respiratory quinones are extracted from freeze-dried cell material using hexane, purified by a silica-based solid phase extraction, and analyzed by HPLC recording absorption spectra (Vieira et al., 2021). For relative quantification, 270 nm for ubiquinones and 326 nm for menaquinones are used.

### 5.9. Propagation of isolated HOB in the reactor and extraction of hydroxyectoine

The isolate was precultured in 20 mL sterilized medium in a glass vial under an H_2_/CO_2_/O_2_ atmosphere (80:10:10) at 30°C for 2 days. Subsequently, 10 mL of the preculture was transferred into a freshly prepared HBI system reactor and incubated as described above. The bacterial cells were harvested via centrifugation. To extract hydroxyectoine, 200 μL of bidistilled water was added for every 10 mg of biomass. The hydroxyectoine concentration was determined following a method described in Laryea et al. (1998). The supernatant (extract) was recovered via centrifugation at 10,000 g for 5 min, and 50 μL of the extract was mixed with 50 μL of 100 mM KH_2_PO_4_, which was further mixed with 900 μL of the labeling solution (2.5 mM 18-crown-6 and 50 mM 4-bromophenacyl bromide in acetonitrile). The labeling reaction was performed for one hour at 80°C. The mixture was vortexed and centrifuged at 1000 g. 200 μL of the supernatant containing the phenacyl esters of hydroxyectoine was directly injected into HPLC (LC-20 AD, Shimadzu) equipped with a Supelcosil LC-SCX column (Sigma-Aldrich, United States). The mobile phase was 22 mmol/L choline in 900 mL/L acetonitrile and 100 mL/L water at a 1.5 mL/min flow rate. The sample was eluted isocratically over twenty minutes and monitored at 254 nm and 28°C. To prepare the standard curve, hydroxyectoine (Sigma-Aldrich) was used at concentrations of 500, 100, 20, and 4 μM.

## Data availability statement

The datasets presented in this study can be found in online repositories. The names of the repository/repositories and accession number(s) can be found at: the raw sequence reads of the 16S rRNA gene amplicons data: https://www.ddbj.nig.ac.jp/DRA016142; the complete genome sequences of A. xylosoxidans strain H1_3_1 and M. mageritense strain H4_3_1: https://www.ddbj.nig.ac.jp/AP028040 and AP027452 the raw reads of genomes: https://www.ddbj.nig.ac.jp/ DRA016141 and DRA015904; the partial 16S rRNA gene sequences of strains H1_3_1 to H4_3_16: https://www.ncbi.nlm.nih.gov/genbank/genbank/, OQ832702 OQ832714.

## Author contributions

XF: Writing – original draft, Writing – review & editing, Investigation, Formal analysis, Methodology, Software, Visualization. DK: Investigation, Writing – review & editing. SH: Writing – review & editing. HN: Writing – review & editing, Methodology, Supervision. TH: Methodology, Supervision, Writing – review & editing. TT: Supervision, Writing – review & editing, Conceptualization. KS: Supervision, Writing – review & editing, Project administration. HK: Supervision, Writing – review & editing, Conceptualization, Writing – original draft.

## Conflict of interest

The authors declare that the research was conducted in the absence of any commercial or financial relationships that could be construed as a potential conflict of interest.

## Publisher’s note

All claims expressed in this article are solely those of the authors and do not necessarily represent those of their affiliated organizations, or those of the publisher, the editors and the reviewers. Any product that may be evaluated in this article, or claim that may be made by its manufacturer, is not guaranteed or endorsed by the publisher.
