# Supplementary material for: Enrichment of halotolerant hydrogen-oxidizing bacteria and production of high-value-added chemical hydroxyectoine using a hybrid biological–inorganic system
Source: Front Microbiol. 2023 Aug 29;14:1254451. doi: 10.3389/fmicb.2023.1254451 (PMC10497747; doi:10.3389/fmicb.2023.1254451)
Supplement: Supplementary file 1 [file Data_Sheet_1.PDF]

Supplementary Material

1 Supplementary Figures

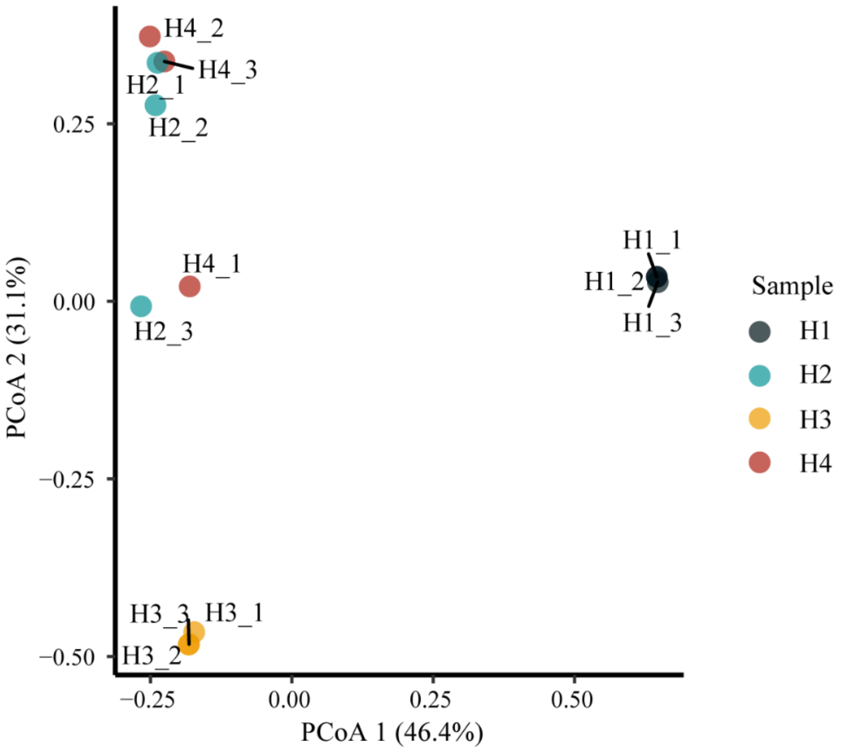

Supplementary Figure 1. PCoA based on Bray–Curtis distances between samples according to inoculum source ($P < 0.05$, PERMANOVA).

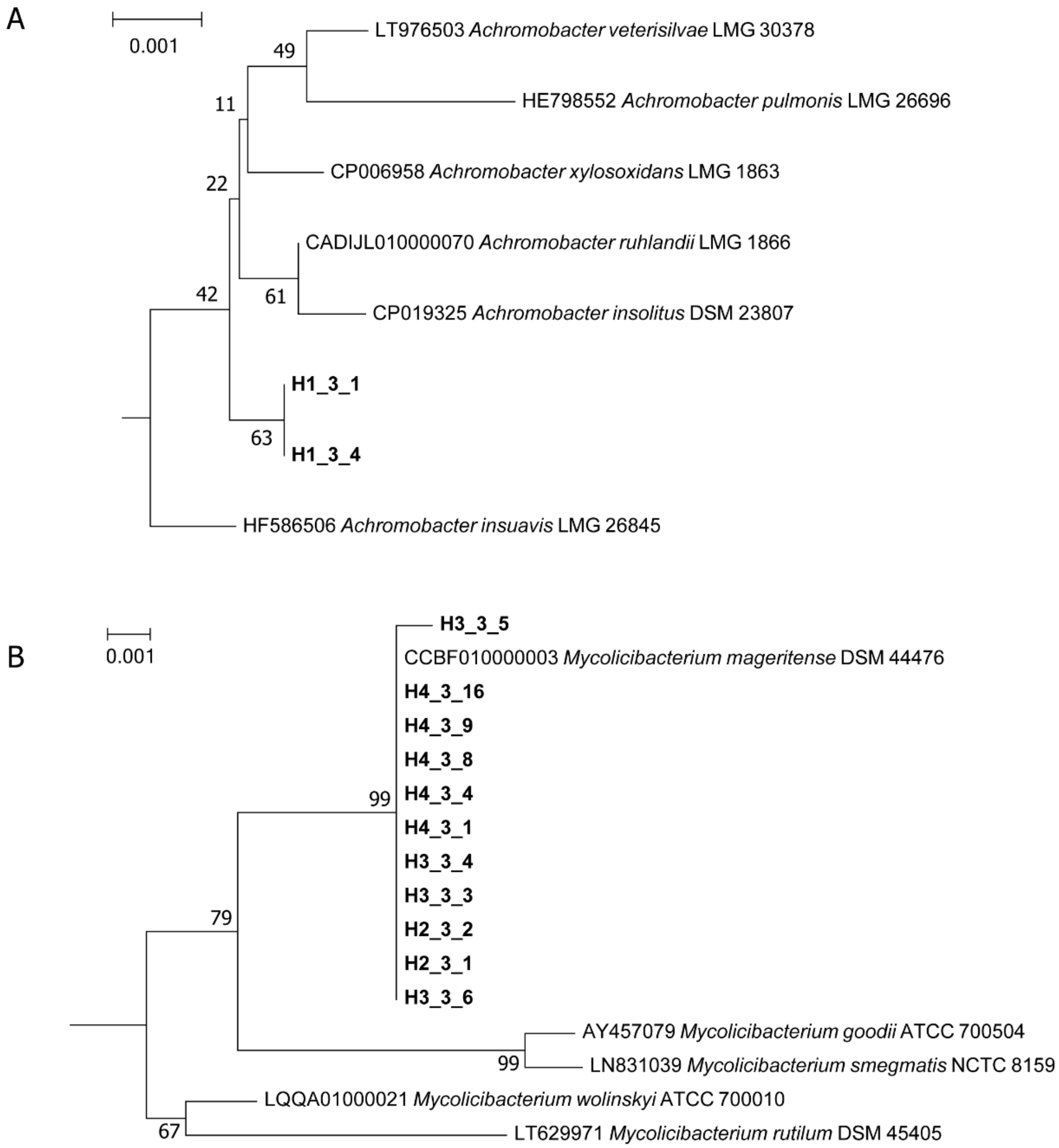

Supplementary Figure 2. Phylogenetic tree illustrating the relationships between 16S rRNA genes of the strains isolated from the HBI reactors of the third enrichment cycle, (**A**) *Achromobacter* species and (**B**) *Mycolicibacterium* species. The evolutionary distances were computed using the Neighbor-joining method based on the Tamura-Nei model in MEGA 7. The percentage of replicate trees in which the associated taxa clustered together in the bootstrap test (2000 replicates) are shown next to the branches. *Bordetella pertussis* Tohama I (BX470248, for *Achromobacter*) and *Mycobacterium tuberculosis* H37Rv (AL123456, for *Mycolicibacterium*) was used as the outgroup (not shown).

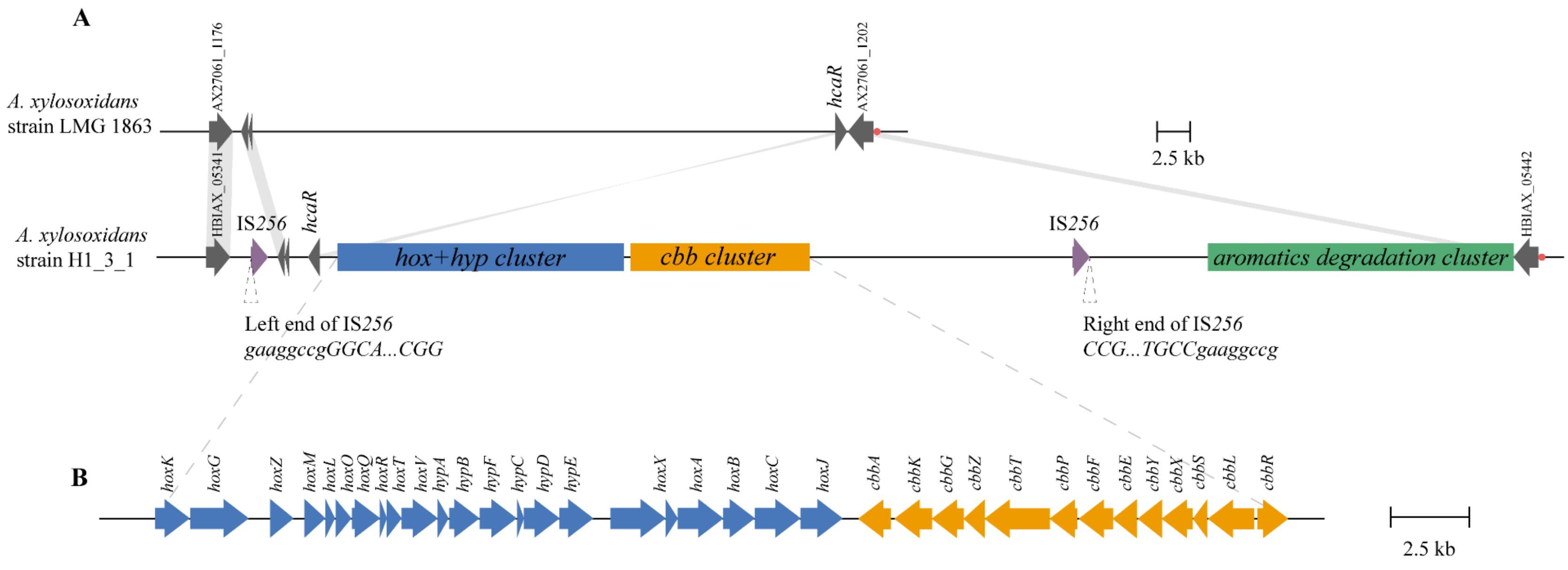

Supplementary Figure 3. The detailed structure of the insertion of *hyp-hox-cbb* cluster in the genome of *A. xylosoxidans* strain H1_3_1 (AP028040) and comparison with the *A. xylosoxidans* type strain LMG 1863 (GCF_000508285.1). (**A**) gene arrangements comparison between *A. xylosoxidans* strain H1_3_1 and the *A. xylosoxidans* strain LMG 1863; (**B**) *hox-hyp* genes (blue arrows) and *cbb* genes (orange arrows) arrangements in *A. xylosoxidans* strain H1_3_1. Other genes were coded as purple arrows, insertion sequences IS*256*; grey sequences, genes could be aligned between strains; red dots, tRNA-Gly. Genes that could not be aligned were not shown.

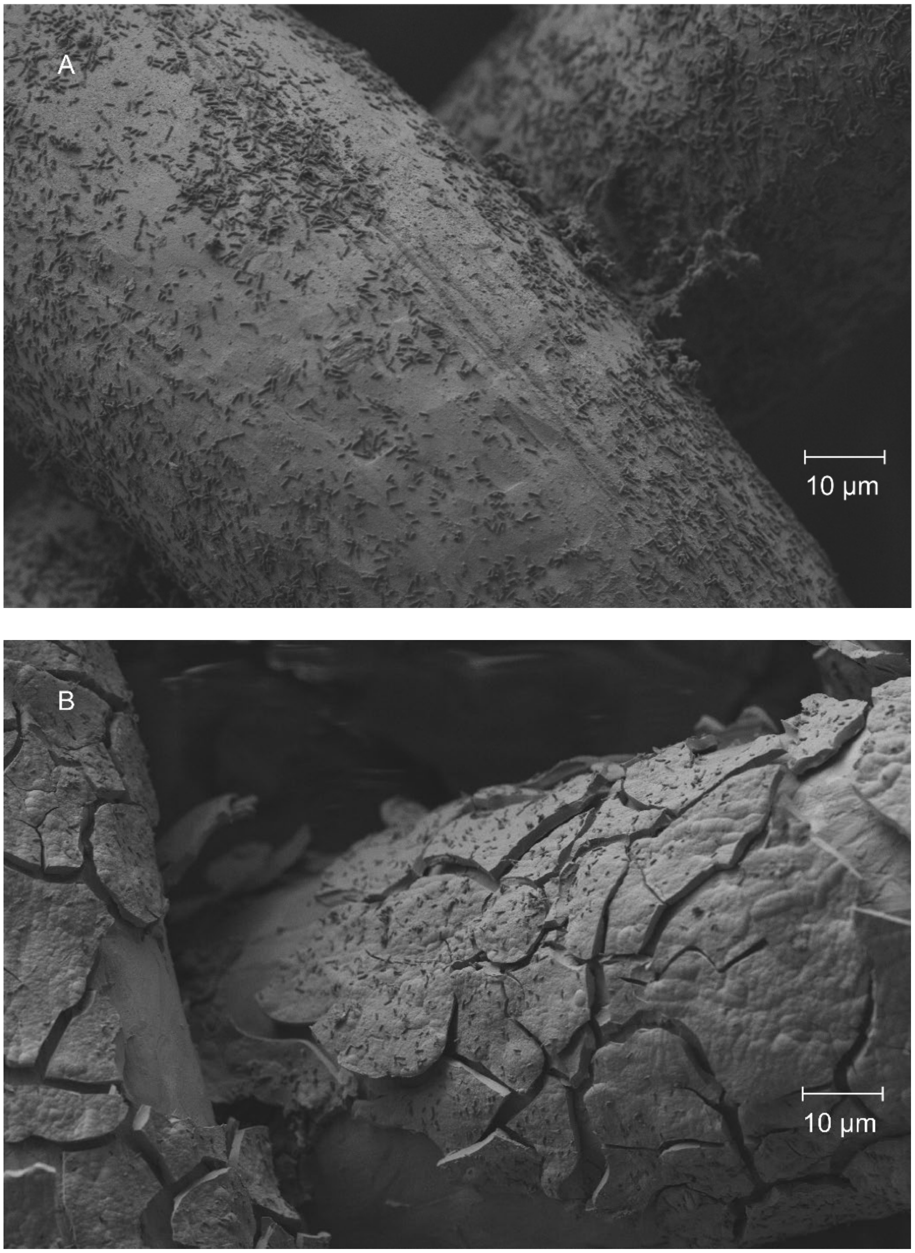

Supplementary Figure 4 Scanning electron microscope image (bar, 10 μm) of the electrode surface after *A. xylosoxidans* strain H1_3_1 grown in a high-ionic-strength medium containing180 mM phosphate buffer for one week at 30°C with a 2.0 V constant voltage supplied. (**A**) cathode surface; (**B**) anode surface.

2 Supplementary Tables

Supplementary Table 1 Fatty acid composition of average composition of *A. xylosoxidans* LMG 1863 (data from Vandamme et al., 2013) and *A. xylosoxidans* strain H1_3_1.

	H1_3_1	LMG 1863
$C_{12:0}$ 2-OH	2.85	3.55 ± 1.08
$C_{14:0}$	1.94	2.56 ± 0.80
$C_{14:0}$ 2-OH	2.68	2.24 ± 1.19
$C_{16:0}$	33.71	32.24 ± 1.95
Cyclo-$C_{17:0}$	9.53	2.79 ± 1.47
$C_{18:0}$	2.74	1.83 ± 1.60
$C_{18:1}$ ω7c	6.83	8.22 ± 2.34
Summed feature 2[a]	9.04	10.32 ± 3.01
Summed feature 3[b]	27.31	34.70 ± 1.13

[a] Summed feature 2: unknown ECL 10.928 and/or iso-$C_{16:1}$ I and/or $C_{14:0}$ 3-OH

[b] Summed feature 3: $C_{16:1}$ *ω7c* and/or $C_{16:1}$ *ω6c*

Vandamme, P., Moore, E.R., Cnockaert, M., De Brandt, E., Svensson-Stadler, L., Houf, K., et al. (2013). *Achromobacter animicus* sp. nov., *Achromobacter mucicolens* sp. nov., *Achromobacter pulmonis* sp. nov. and *Achromobacter spiritinus* sp. nov., from human clinical samples. *Syst. Appl. Microbiol.* 36(1), 1-10. doi: 10.1016/j.syapm.2012.10.003.

Supplementary Table 2 Phenotypic characteristics of *A. xylosoxidans* LMG 1863 (data from Vandamme et al., 2013) and *A. xylosoxidans* strain H1_3_1.

	LMG 1863	H1_3_1
Nitrate reduction	+	+
Assimilation of glucose	+	+
Assimilation of maltose	-	-
Assimilation of potassium gluconate	+	+
Assimilation of capric acid	+	+
Assimilation of adipic acid	+	+
Assimilation of malate	+	+
Assimilation of phenylacetic acid	+	+
Acid production from glucose	-	-
Assimilation of arabinose	-	-
Assimilation of mannose	-	-
Oxidase	+	+
Activity of alkaline phosphatase	+	+w
Activity of butyrate esterase (C4)	+	+
Activity of valine arylamidase	-	-
Activity of acid phosphatase	+	+
Activity of phosphoamidase	-	+w

Supplementary Table 3 EIS of the HBI reactor under the low-ionic-strength or high-ionic-strength condition. The solution resistance (R_{ohm}), the charge transfer resistances of the anode and cathode (R_{act_anode} and $R_{act\_cathode}$), and the total internal resistance (R_{int}) were determined.

Medium type	R_{ohm} (Ω)	R_{act_anode} (Ω)	$R_{act\_cathode}$ (Ω)	R_{int} (Ω)
Low-ionic-strength medium	76	39	214	329
High-ionic-strength medium	21	25	96	142
